# A rare case of PAPASH syndrome with a NOD2 mutation

**DOI:** 10.1016/j.jdcr.2026.04.032

**Published:** 2026-04-23

**Authors:** Yaelle Shaked, Anika Pulumati, Alison H. Kucharik, Kayla Fourzali, Catherine Nicole Hawkins, Meredith Thomley

**Affiliations:** a Icahn School of Medicine at Mount Sinai, New York City, New York; b Department of Dermatology and Cutaneous Surgery, University of South Florida Health Morsani College of Medicine, Tampa, Florida

**Keywords:** autoinflammatory syndromes, neutrophilic dermatosis, PAPA, PAPASH, PASH, pyoderma gangrenosum

## Case

A 40-year-old female with a past medical history of polysubstance use, seronegative arthritis, type 2 diabetes, and hidradenitis suppurativa (HS) presented to the emergency room with a worsening ulcer on the left foot. She reported swelling and purulent drainage at the site following minor trauma. During her admission, the patient underwent an incision and drainage with wash out and bone biopsy of the left foot. Progressive worsening of the wound, as well as the development of multiple distant ulcerations, prompted Dermatology consult to evaluate for pyoderma gangrenosum (PG) in the setting of seronegative arthritis and HS.

**Fig 1:**
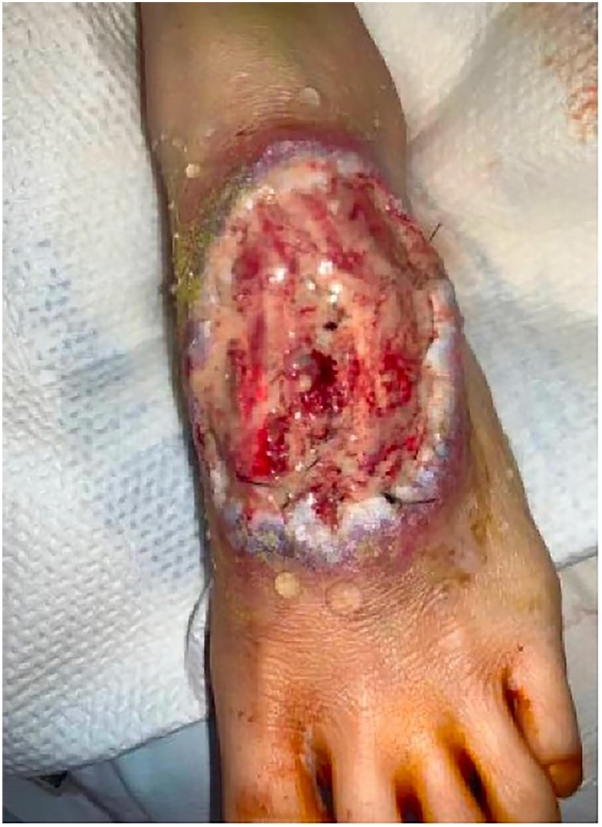
Left dorsal foot, exudative ulcer with violaceous, undermined borders and exposed tendon.

**Fig 2:**
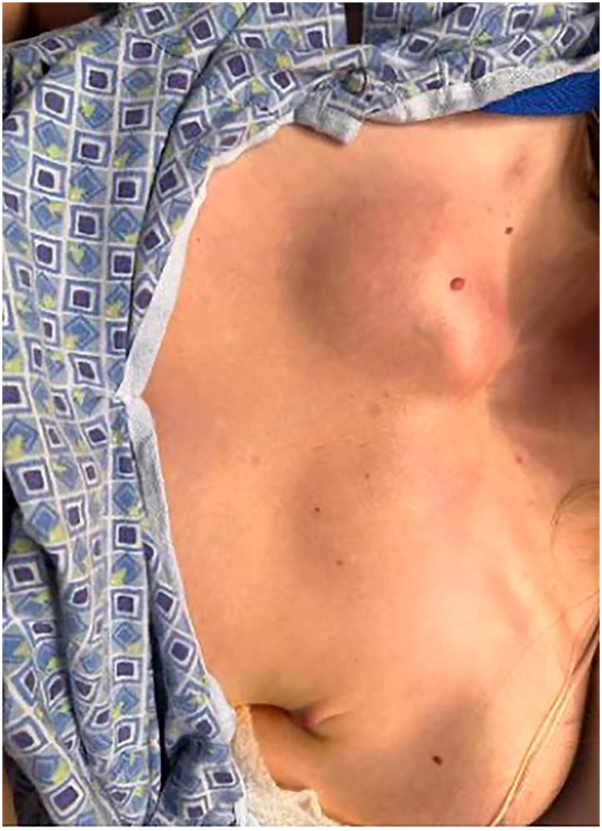
Upper chest, 2 fluctuant subcutaneous nodules.

**Fig 3:**
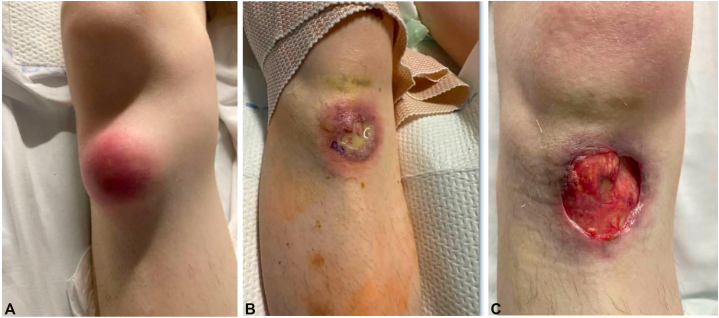
**A,** Right knee, erythematous subcutaneous nodule. **B,** Right knee, violaceous ulcer with an undermined border and purulent drainage. **C,** Right knee, status post 2 doses of infliximab 5 mg/kg and prednisone 60 mg.

Physical examination revealed an exudative ulcer with violaceous, undermined borders and exposed tendons on the left dorsal foot (Fig 1). Similar ulcerations were noted on the left upper arm and bilateral inguinal creases. Additionally, the patient had bilateral tender, fluctuant subcutaneous nodules on the upper chest that did not ulcerate (Fig 2). Throughout the admission, the patient developed erythematous, tender subcutaneous nodules on the right knee and left anterior shin that subsequently ulcerated with violaceous, undermined borders and purulent drainage (Fig 3, *A* and *B*). In the bilateral axillae were nontender, cicatricial sinus tracts, and double-headed comedones, consistent with HS. Two punch biopsies were taken from the periphery of the right knee ulcer and sent for H&E and microbial cultures (bacterial, fungal, acid-fast bacilli, and nocardia). Genetic testing was ordered due to the history of HS and seronegative arthritis, now presenting with concern for multifocal pyoderma gangrenosum.

Biopsy showed a dense neutrophilic infiltrate with admixed lymphocytes and plasma cells. All infectious evaluations including special stains (PAS, FITE, and Gram) on skin histopathology, as well as wound, tissue, and bone biopsy cultures were all negative, supporting the noninfectious etiology PG. Laboratory workup was unremarkable, including negative HIV antigen/antibody, hepatitis B and C serologies, and QuantiFERON-gold. Genetic testing was evaluated for the PSTPIP1, MEFV, NLRp3, and NOD2 genes. The c.2722G>C(p.Gly908Arg), an increased risk allele in the NOD2 gene, was identified. Colonoscopy ruled out inflammatory bowel disease. Given the patient’s history of HS, seronegative arthritis, nodules progressing into ulcers, skin biopsy pathology, and genetics results, the patient was diagnosed with PAPASH (pyogenic arthritis, PG, acne, and HS) syndrome. The patient was started on infliximab 5 mg/kg at weeks 0, 2, and 6, initiated while inpatient, followed by maintenance infusions planned for every 8 weeks and prednisone 60 mg daily with a 5 mg taper weekly. After the initial 2 doses of infliximab, the patient showed significant improvement (Fig 3, *C*). Due to concern for pathergy, invasive management of the subcutaneous nodules was avoided, and supportive wound care with topical gentamicin and clobetasol ointment was recommended.

## Discussion

PG is a rare neutrophilic dermatosis that commonly affects women over 50 y of age with an incidence of 3-10 cases per million annually.^1^, ^2^, ^3^ PG typically presents as pustules or papules that progress into ulcers with a violaceous, undermined border commonly found on the lower extremities. PG was once considered a diagnosis of exclusion until the 2018 DELPHI criteria, which established a set of 1 major and 8 minor criteria to confirm the diagnosis.^3^ Our patient met the major criterion (biopsy of the ulcer edge presenting with neutrophilic infiltrate) and 5 of 8 minor criteria including, exclusion of infection, pathergy, erythematous undermined border with tenderness at ulceration site, multiple ulcers (one of which was present on the lower extremity), and decreased ulcer size after 1 month of initiating immunosuppressive medications.^3^ While classically found in association with systemic diseases such as IBD, inflammatory arthritis, and solid organ malignancies, PG can also be considered as part of a subset of autoinflammatory syndromes including PAPA (pyogenic arthritis, PG, and acne), PASH (PG, acne and HS), or PAPASH.^2^^,^^4^

Both neutrophilic dermatosis and autoinflammatory syndromes are thought to result from innate immune system overactivation, particularly involving an increase in IL-1.^4^ This causes the release of cytokines like tumor necrosis factor alpha and interferon gamma, leading to neutrophil recruitment and activation.^4^ On histopathology, this typically presents with a dense neutrophilic infiltrate without signs of infection. Mutations in PSTPIP1, MEFV, NOD2, and NLRP3 genes have been associated with PAPA and PASH syndromes.^5^, ^6^, ^7^ While Marzano et al^8^ reported a PSTPIP1 mutation in association with PAPASH syndrome, the genetic profile of PAPASH remains less defined than that of PAPA or PASH. Due to the increased release of IL-1ß and tumor necrosis factor alpha, common treatment approaches include the use of tumor necrosis factor alpha inhibitors, IL-1 inhibitors, and topical or systemic corticosteroids.^4^^,^^7^ The patient was started on infliximab and systemic steroids, demonstrating significant improvement after the initial doses of infliximab. The patient’s multifocal ulcers all decreased in size and became less painful, and 1 of the 2 subcutaneous nodule on the chest significantly decreased in size.

While PAPA and PASH syndromes have been described in the literature, there are a limited number of case reports describing PAPASH syndrome. While acne is considered a defining component of PAPASH syndrome, increasing recognition of phenotypic heterogeneity in autoinflammatory syndromes, specifically PAPA, PASH and PAPASH, suggests that patients may present with incomplete or variable clinical findings. Although an alternative classification, such as PASH with associated seronegative arthritis could be considered, the coexistence of PG, HS, and inflammatory arthritis in the setting of a NOD2 variant raises the possibility that genetic modifiers may be contributing to the phenotypic variability.

To our knowledge, this is one of the first reported cases of PAPASH presenting without acne in the context of a NOD2 variant, highlighting the broader phenotypic spectrum of PAPASH syndrome. The NOD2 mutation is frequently associated with autoinflammatory conditions like Crohn’s disease, in which it promotes neutrophil recruitment through hyperactivation of the NF-κB pathway.^7^ In this case, no evidence of Crohn’s disease was found on colonoscopy, suggesting the NOD2 mutation may play a role in the phenotypic spectrum of PAPASH. Recognition of such atypical presentations emphasizes the importance of considering PAPASH even when all classic features are not present. Future studies may further characterize the full phenotypic spectrum of PAPASH and elucidate genotype-phenotype correlations to more effectively tailor treatment options for patients.

## Conflicts of interest

None disclosed.
